# Chloro Nitrous Oxyd

**Published:** 1869-12

**Authors:** 


### CHLORO NITROUS OXYD.

BY DR. PARKER.

An article in the July No., of the Dental Register, by R. N. Lawrence, D. D. S., in which chloro nitrous oxyd is disposed of so confidently, is of such a very peculiar nature that I wish to say a few words in reply.

In the first part of May last, I received a letter of which the following is an exact copy, excepting the introductory clause:

"I am using Nitrous Oxyd and have been for a long time I have felt all the inconveniences referred to by Dr. Parker, I would ask is your manner of producing it a secret or *patented*, if not, I would like to know how it is done and would be pleased to hear from you at your earliest convenience."

I am very Respectfully Yours,

R. N. Lawrence, D.D.S.

I was not favorably impressed by his allusion to patents, but remembering that every man *must* have his hobby, I wrote him "that it was not a secret but was *patented*, but was none the less valuable on that account, and as I knew of no good reason why a man should deprive himself of the use of a good thing merely because it was patented, I should answer his enquiry the same as though it was not *patented*," and then gave him minute directions how to make chloro nitrous oxyd, after the manner that over a year's experience in its use and a large number of careful experiments had taught me as being the best; and the only "pay" that I asked of him for "imparting the great secret" was that he should give it a fair trial and report his success to me, whatever it might be, I had reason to expect that if he attempted it at all he would follow my directions with an exactness that the importance of the subject and the safety of his patients demanded, but instead of that he proceeded, as he says, "to try its merits" by not following my directions in a single particular, and then reported his experience through the Register with a confidence that I think is not justified by the nature and extent of his investigations. He made a blunder, not a very dangerous one to be sure, but a very unhappy one, by using eight times the proportional amount of alcohol that I directed him to use, and my experience has taught me that a much less proportional amount than he used will frequently produce after effects similar to those produced by chloroform. I can not conceive why the subject of patents was lugged into that article. "Is the value of chloro nitrous oxyd to be decided by it." "Has its safety or efficiency any connection with that subject whatever." I hope it was not intended to beget in the minds of others that prejudice which evidently pervaded his own. The subject of patents I will not discuss here, but will say, let us act like men, and not "wrap the mantle of selfishness about us," and say, "sir," give me the benefit of your time and money spent in developing this thing, or I will treat you as a man outside the pale of common civility. After all, Dr. Lawrence just barely missed of success. An escape so narrow, under such circumstances, speaks volumes in favor of the inherent virtues of chloro nitrous oxyd.

No man ever did, or ever will, attain to any degree of success with nitrous oxyd, if he attempts it with the same spirit and lack of skill displayed in this case. Indeed who would speak so confidently after such a trial with it, and what would such an experience be considered worth to the profession. He says that he produced anæsthesia in much less time, it was more profound and more prolonged, and recovery was not as speedy as when nitrous oxyd alone was used, the same results, as far as they go, as were claimed in the article written by Dr. Parker. The after effects that he complains of were not the legitimate results of chloro nitrous oxyd. Had he communicated with me, I think he would have had no further trouble on that score, and then he could have said also, that it was safe and pleasant, thereby completing the catalogue. The causes that produce congestive chills in different persons are so various that I will not venture an opinion as to what caused them in his sixth case. I am acquainted with a lady who can not smell of creosote without producing congestive chills. As to his experiments with the lady troubled with heart disease, when he had reason to think that he was "putting to trial the fearful issues of life or death," I have nothing to say. With me chloro nitrous oxyd is not an experiment. I have used it a year and a half with all kinds of subjects and under all the various circumstances that the practice of Dentistry will develop, and have found it *safe, pleasant and reliable.* I have nothing to say against nitrous oxyd, I appreciate its benefits to the profession and have felt all its weakness. If its usefulness can be increased it is certainly desirable. If chloro nitrous oxyd promises to do so, let us lay aside *all* prejudices and give it an impartial and exhaustive trial. Let us bring all the light attainable to bear in our investigations, and then let it stand or fall on its own merits. I do not expect that every one will succeed with it. The history of the introduction of nitrous oxyd would seem to indicate that such a result is not possible; neither is it claimed that it is *all* that we desire in an anæsthetic agent, but my experience satisfies me that it is the best now in use in Dental surgery.

I shall continue to give directions how to make chloro nitrous oxyd, in answer to the enquiries of those who wish it, on condition that they give it a fair trial and ıeport their success to me.

